# Assessment of Adverse Drug Events, Their Risk Factors, and Management Among Patients Treated for Multidrug-Resistant TB: A Prospective Cohort Study From Pakistan

**DOI:** 10.3389/fphar.2022.876955

**Published:** 2022-05-17

**Authors:** Farman Ullah Khan, Amjad Khan, Faiz Ullah Khan, Khezar Hayat, Asim ur. Rehman, Jie Chang, Waseem Khalid, Sidra Noor, Asad Khan, Yu Fang

**Affiliations:** ^1^ Department of Pharmacy Administration and Clinical Pharmacy, Xi’an Jiaotong University, Xi’an, China; ^2^ Center for Drug Safety and Policy Research, Xi’an Jiaotong University, Xi’an, China; ^3^ Shaanxi Center for Health Reform and Development Research, Xi’an Jiaotong University, Xi’an, China; ^4^ Research Institute for Drug Safety and Monitoring, Institute of Pharmaceutical Science and Technology, China’s Western Technological Innovation Harbor, Xi’an, China; ^5^ Department of Pharmacy, Quaid-i-Azam University, Islamabad, Pakistan; ^6^ Institute of Pharmaceutical Sciences, University of Veterinary and Animal Sciences, Lahore, Pakistan; ^7^ Programmatic Management of Drug-Resistant TB Site Pakistan Institute of Medical Sciences, Islamabad, Pakistan; ^8^ Pharmaceutical Sciences, Universiti Sains, Malaysia, Malaysia; ^9^ School of Pharmaceutical Sciences, Universiti Sains, Malaysia

**Keywords:** multidrug-resistant tuberculosis, adverse drug event, management, pharmacist, patient satisfaction

## Abstract

**Background:** Multidrug-resistant tuberculosis (MDR-TB) is a growing public health problem. Treatment regimens used against MDR-TB are costly, prolonged, and associated with more side effects as compared with the drug-susceptible tuberculosis. This study was framed to determine the incidence of adverse drug events, risk factors, and their management in MDR-TB patients.

**Methods:** This prospective follow-up cohort study was conducted at the site of programmatic management of drug-resistant TB located at the Pakistan Institute of Medical Sciences, Islamabad. All patients, irrespective of their age, gender, and ethnicity, were included in the study. Adverse drug events were observed in patients at different time points during the study. Patients enrolled for the treatment from January 2018 were prospectively followed till December 2020 up to their end treatment outcomes.

**Results:** Out of 126 MDR-TB patients enrolled for treatment, 116 met the inclusion criteria and were included in the final analysis. Most patients (50.9%) were between 18 and 45 years of age. A minimum of one adverse event was experienced by (50.9%) patients. Of all the adverse events, gastrointestinal disorders were more frequent (47.4%), followed by arthralgia (28.4%) and psychiatric disturbance (20.6%). Furthermore, multivariate analysis showed a significant association with the incidence of adverse events in patients with age group above 60 years (odds ratio (OR) 4.50; 95% CI 1.05-19.2), active smokers (OR 4.20; 95% CI 1.31-13.4), delayed reporting to the TB center (OR 4.03; 95% CI 1.34-12.1), and treatment without bedaquiline regime (OR 3.54; 95% CI 1.23-10.1). Most of the patients (94.6%), counseled by the pharmacist, were found to be satisfied with the information provided and looked for more pharmacist counseling opportunities in the management of MDR-TB.

**Conclusion:** Current findings recommend that ADEs might be well managed by timely identification and reporting. Bedaquiline coupled with other active medications lowered the chance of ADEs in MDR-TB patients. Elderly patients, active smoking behavior, and those who have a delay in the treatment initiation are more prone to ADEs. Clinical pharmacist’s contribution to TB control programs may help caregivers and patients concerning the rational use of medication, early detection, and management of ADEs.

## Introduction

The emergence and spread of multidrug-resistant tuberculosis (MDR-TB) poses significant challenges to the control and successful eradication of TB, particularly in developing countries (Iradukunda et al., 2021). Despite all the efforts made throughout the world by the WHO, the treatment success rate for MDR-TB is still low. The WHO targets a success rate of at least 75% for MDR-TB patients; however, the most recent statistics on treatment outcomes indicate a success rate of 56% (Baluku et al., 2021; Yang et al., 2017). This is mainly because the MDR-TB therapy is costly, has low efficacy, and has more adverse drug events when compared to drug-susceptible TB (Baluku et al., 2021). One of the factors causing MDR-TB is non-compliance to the anti-MDR-TB treatment regimen. The most important independent factor underlying non-compliance to treatment is adverse events of medications (Resende and Santos-Neto, 2015; Merid et al., 2019; Sankar et al., 2021). The overall incidence of ADEs caused by anti-TB medications ranges from 5.1% to 83.5% in different populations (Kefale et al., 2020). ADEs increase patient suffering and increase substantial additional costs because of added outpatient visits, laboratory investigations, and hospitalizations, in more serious instances (Resende and Santos-Neto, 2015). Despite these difficulties, up to 80% of the treatment results can be achieved by effectively treating MDR-TB patients. Most ADEs can be controlled with over-the-counter and commonly prescribed medications. If the ADEs are mild, then proceeding with supplementary medications may be helpful (Trubnikov et al., 2021). In some cases, ADEs disappear over time and patients should be encouraged to tolerate them until the effects subside. Studies have shown that multidrug regimens can cause numerous ADEs such as nausea, vomiting, diarrhea, skin reaction, ototoxicity, peripheral neuropathy, psychiatric symptoms, nephrotoxicity, impaired vision, and hypothyroidism (Bezu et al., 2014; Merid et al., 2019).

Pakistan ranked the highest for MDR-TB burden in EMRO (Eastern Mediterranean Region of the WHO) and fourth highest country in the world (WHO, 2018; NTP, 2020). Drug-resistant tuberculosis is becoming more common in Pakistan, which highlights the need for efficient treatment regimens for MDR/XDR-TB (Javaid et al., 2018). Despite being the country with the highest burden of MDR-TB in EMRO, there is little information available about the management of adverse effects and treatment outcomes of patients with MDR-TB in Pakistan. To attain the 2025 goal of complete elimination of TB, a better understanding of the ADE of all drugs used to treat MDR-TB is an important and much-needed step in improving patient management and treatment outcomes. Therefore, close monitoring of patients is important to ensure that the ADEs are timely reported and managed. Clinical pharmacists providing pharmaceutical care services have been shown to improve adherence to therapy and reduce potential ADEs. More data on their characteristics and management are very valuable for clinicians and pharmacists. Therefore, this study was framed to determine the incidence, risk factors, and management of adverse events encountered in clinical practice.

## Materials and Methods

### Study Site

This prospective case series analysis was carried out at the unit for programmatic management of drug-resistant TB (PMDT) located in the Pakistan Institute of Medical Sciences, Islamabad. Doctors, nurses, data operators, coordinators, pharmacists, psychologists, and other support personnel work at the research site to help MDR-TB patients achieve a treatment success rate of 75%.

### Study Population and Eligibility Criteria

Patients enrolled for the treatment from January 2018 were followed from the baseline visit. Those registered before 2018 and still on treatment were retrospectively followed till January 2018. Afterward, all the enrolled patients were prospectively followed till December 2020 up to their end treatment outcomes. All MDR-TB patients, irrespective of their age, gender, and ethnicity, were included in the study, while the multidrug-resistant cases those were transferred out from the study center or were still on the treatment medications during the final day of analysis were excluded.

### Follow-Up and Adverse Events Monitoring

Based on WHO guidelines for the treatment of MDR-TB and previous research, a standardized data-collecting form was developed. The supervisory committee and healthcare personnel at the study site also provided input. The patients were first assessed for symptoms at baseline and then reviewed and evaluated on a monthly basis by the doctor. Once the screening tests at baseline were completed, the MDR-TB patients were enrolled to be treated with the treatment regimen according to the NTP guidelines. All the drugs were provided at their maximum prescribed dosage, which were determined by the patient body weight. The treatment of all the patients after sputum culture conversion was continued for a minimum of 18 months. The patients were closely monitored during the follow-up visits to assess the ADEs Table 1. Specialist clinicians, pharmacists, and data coordinators reviewed every patient once a month to make sure their treatment was as effective as possible. All the patients were being assessed on a monthly basis and were treated as outpatients. To ensure adherence to the treatment, the patients were monitored by trained treatment supporters.

**TABLE 1:** Definition of side effects.

Gastrointestinal disorders	Incidence of nausea, abdominal pain, vomiting, anorexia, sour taste in the mouth, flatus and cramping, diarrhea, epigastric burning or discomfort, hematemesis, melena, and positive analysis by endoscopic study
Psychiatric disturbances	Psychosis symptoms, anxiety, nightmares, delusions, suicidal ideation, depression, and visual or auditory hallucinations
Dermatologic conditions	Every skin change includes severe generalized rash, itch, bronzing, and photosensitivity reaction
Peripheral neuropathy	Peripheral neuropathy was defined as a MDR-TB patient referring to damage to the nerves located outside of the central nervous system, signs of numbness, and tingling or burning sensation in the extremities diagnosed by the physician or electromyography
Hepatotoxicity	Any elevation with or without symptoms of serum transaminases or serum bilirubin greater than 3 or 5 times than normal upper limit
Hearing disturbance	Hearing loss and signs of auditory toxicity and tinnitus
Visual impairment	Pain on moving the eye, visual changes, and vision loss
Arthralgia	Serum uric acid levels may be raised, aching around the joints, pain, and swelling in the joints

Patients found resistant in the Xpert MTB/RIF diagnostic test were registered as MDR-TB cases and treated with a regimen proposed by the WHO and National TB guidelines (WHO, 2012; WHO, 2014). Baseline laboratory tests, such as complete blood count, hepatitis, HIV, blood sugar level, kidney and liver function tests, urine routine test, and thyroid function test were conducted prior to the start of the treatment. AEs were recorded after laboratory test confirmation, and only those AEs that had at least one abnormal value were considered significant. As a requirement of PMDT, on each visit, ADEs were documented based on laboratory results, self-recorded by patients, or monitored by doctors and pharmacists. All patients were assessed for scheduled follow-up visits. Moreover, free laboratory tests and medications were provided, and both patients and treatment supporters were provided with nutritional and transport allowance support**.**

During corona virus infectious disease (COVID-19) pandemic, protocol of the National TB Control Program (NTP) for the protection from COVID-19 was strictly followed for both patients and healthcare professionals (NTP, 2020). According to the instruction of the NTP, an isolated triage area was assigned for all patients to separate them from suspected and non-suspected COVID-19 patients. Appropriate digital and telecommunication support were provided to the patients to ensure communication if they want any consultation for the management of ADEs caused by anti-MDR-TB treatment.

### Operational Definitions

According to WHO guidelines, treatment outcomes are classified as successful when the TB patient finalizes the treatment medication to indicate cure and completion, while unsuccessful treatment outcomes are classified as treatment failure, treatment defaulters, and died patients (WHO, 2021). This study assessed patients’ satisfaction with pharmacist counseling services. A novel tool known as the patient satisfaction feedback on counseling (PSF) questionnaire in English and Urdu languages was used to document the satisfaction of patients following pharmacist counseling sessions (Naqvi et al., 2019).

### Statistical Analysis

Distributions of participants’ characteristics and the cumulative incidences of adverse events were studied using descriptive statistics. Variables having significant association in the chi-square test were further analyzed through a univariate and then a multivariate logistic regression model. Variables with a *p*-value of <0.15 in the univariate regression analysis were analyzed in the final multivariate regression model. The occurrence of substantial intercorrelations between two or more independent variables in a regression model is referred to as multicollinearity. We examined the collinearity and tolerance value for each variable while creating the multivariate binary logistic regression. If the variables have a strong correlation (variance inflation factor = 10 and tolerance value > 0.1), one of them was excluded from the concluding model (Pallant, 2020). The final multivariate binary logistic regression model was adjusted using the Hosmer–Lemeshow test. The odds ratios with 95% confidence intervals were considered to measure the significant (*p* ≤ 0.05) relationship between variables and adverse events. We used the Statistical Package for the Social Sciences, version 26 (SPSS) in all statistical analyses.

## Results

### Baseline Characteristics

Overall, 126 MDR-TB patients signed up for the treatment at the site during data collection for this study. A total of ten patients were omitted from the trial because they did not match the study’s eligibility requirements. A total of 116 MDR-TB patients were included and followed for their therapeutic outcomes. Baseline clinical and sociodemographic characteristics of the patients are given in Table 2.

**TABLE 2:** Baseline demographic and clinical characteristics of the participants (n, 116).

Variable	Frequency (n %)
Age (years)	
<18	21 (18.1)
19–45	59 (50.9)
>46	36 (31.0)
Gender	
Female	50 (43.1)
Male	66 (56.9)
Marital status	
Married	97 (83.6)
Unmarried	19 (16.4)
Education	
Illiterate (no education at all)	75 (64.7)
Literate (primary and above)	41 (35.3)
Employment	
Employment	10 (8.6)
Unemployment	46 (39.7)
House wife	27 (23.3)
Student	20 (17.2)
Self-employment	13 (11.2)
Monthly income	
<20000	65 (56)
≥20000	51 (44)
Residency	
Rural	77 (66.4)
Urban	39 (33.6)
Smoking	
Active smoking	26 (22.4)
No smoking	91 (78.6)
Baseline weight	
<40 kg	36 (31)
≥40 kg	80 (69)
Comorbidity (30)	
Diabetes	19 (15.5)
Hypertension	7 (6)
Hepatitis	3 (2.5)
HIV	1 (0.9)
Number of resistant drugs	
≥2	59 (50.9)
<2	57 (49.1)
Resistance to SLD drugs	
Yes	28 (24.1)
No	88 (75.9)
Sputum smear	
Negative/scanty	33 (28.4)
Positive	83 (71.6)
Regime containing bedaquiline	
Bedaquiline	32 (27.6)
No bedaquiline	84 (72.4)
Successful treatment outcomes (71)	
Cured	62 (53.4)
Completed	9 (7.8)
Unsuccessful treatment outcomes (45)	
Failure	4 (3.4)
Died	28 (24.1)
Lost to follow up	13 (11.2)
Adverse events	
Adverse event reported	60 (50.9)
Adverse event not Reported	56 (49.1)

SLD, second line drug.

Most patients (50.9%) were between 18 and 45 years of age. Male and female were 80 (56.9%) and 56 (43.1%), respectively. Nearly 66% of the patients resided in rural areas, 64.7% have no formal level of education, and 83.6% patients were married. Active smokers were 21.6%, and 25.8% had one or more comorbidities, and 27.6% were those with regime containing bedaquiline. Most of the patients (69%) had a baseline body weight of above 40 kg.

### Predictors of Adverse Drug Events

A statistically significant association was found between the incidence of ADEs and age, comorbidities, smoking, baseline weight, delayed reporting to the TB center, and regime plan containing bedaquiline. No statistically significant association was identified between gender, marital status, education, employment status, residency, and number of drug resistance (Table 3).

**TABLE 3:** Association between patients’ characteristics and incidence of ADEs (n, 116).

Variable	ADE reported (n, 60)	ADE not Reported (n, 56)	*p*-value
Age (years)			0.01
<18	7 (11.7)	13 (23.2)	
19–45	25 (41.7)	31 (55.4)	
>46	28 (46.6)	12 (21.4)	
Gender			0.21
Female	31 (52.5)	35 (61.4)	
Male	28 (47.5)	22 (39.2)	
Marital status			0.12
Married	53 (88.3)	44 (78.6)	
Unmarried	7 (11.7)	12 (21.4)	
Education			0.39
Illiterate (no education at all)	40 (66.7)	35 (62.3)	
Literate (primary and above)	20 (33.3)	21 (37.7)	
Employment status			0.49
Employment	2 (3.3)	8 (14.3)	
Unemployment	26 (43.3)	20 (35.7)	
House wife	17 (28.3)	10 (17.9)	
Student	10 (16.8)	10 (17.9)	
Self-employment	5 (8.3)	8 (14.2)	
Monthly income			0.14
<20000	37 (61.7)	28 (50)	
≥ 20000	23 (38.3)	28 (50)	
Residency			0.44
Rural	39 (65)	38 (67.9)	
Urban	21 (35)	18 (32.1)	
Smoking			0.01
No smoking	41 (65)	50 (91.1)	
Active smoking	21 (35)	5 (8.9)	
Baseline weight			0.04
<40 kg	24 (40)	13 (23.2)	
≥40 kg	36 (60)	43 (76.8)	
Reported to the TB center			0.00
More than a month delay	22 (36.7)	7 (12.5)	
Less than a month delay	38 (63.3)	49 (87.5)	
Comorbidity			0.04
Yes	20 (33.3)	10 (17.9)	
No	40 (66.7)	46 (82.1)	
Number of resistant drugs			0.13
≥2	27 (45)	32 (57.1)	
<2	33 (55)	24 (42.9)	
Regime containing bedaquiline			0.01
Bedaquiline	11 (18.3)	21 (37.5)	
No bedaquiline	49 (81.7)	35 (62.5)	
Treatment outcomes			0.02
Successful outcome	31 (51.7)	40 (71.4)	
Unsuccessful outcome	29 (48.3)	16 (28.6)	

ADE, adverse drugs events; SLD, second line of drug (*p* ≤ 0.05).

Furthermore, during multivariate analysis, the variables that had a significant association with the occurrence of AEs were patients with age group above 60 years (odds ratio (OR) 4.50; 95% CI 1.05-19.2), active smokers (OR 4.20; 95% CI 1.31-13.4), and delayed reporting to the TB center (OR 4.03; 95% CI 1.34-12.1). Similarly, bedaquiline regime had the lowest incidence of ADEs among amino salicylic acid, linezolid, and injectable drugs (OR 3.54; 95% CI 1.23-10.1). Exploring the impact of ADEs on TB treatment outcomes through univariate analysis, the occurrence of ADEs had a significant risk of positive association with unsuccessful treatment outcomes (OR 2.33; 95% CI 1.08-5.05). However, in multivariate analysis, no level of significance was found Table 4.

**TABLE 4:** Risk factors associated with adverse drugs events.

Variable	ADE reported	COR (95%CI)	AOR (95%CI)
Age (years)			
<18	7 (11.7)	References	References
19–45	25 (41.7)	2.89 [1.22–6.81]	1.95 [0.65–5.78]
>46	28 (46.7)	4.33 [1.38–13.5]٭	4.50 [1.05–19.2]٭
Smoking			
No smoking	41 (65)	References	
Active smoking	21 (35)	5.49 [1.90–15.8] ٭	4.20 [1.31–13.4]*
Baseline weight			
<40 kg	24 (40)	References	References
≥40 kg	36 (60)	2.20 [0.98–4.94]٭	1.80 [0.66–4.92]
Reported to MDR-TB center			
Less than a month delay	22 (36.7)	References	References
More than a month delay	38 (63.3)	4.05 [1.56–10.4]٭	4.03 [1.34–12.1]٭
Comorbidity			
No	20 (33.3)	References	References
Yes	40 (51.7)	2.30 [0.96–5.48]*	1.35 [0.47–3.88]
Regime containing bedaquiline			
Bedaquiline	11 (18.3)	References	References
No bedaquiline	49 (81.7)	2.88 [1.23–6.71]٭	3.54 [1.23–10.1] ٭
Treatment outcomes			
Successful outcome	31 (51.7)	References	References
Unsuccessful outcome	29 (48.3)	2.33 [1.08–5.05]٭	1.81 [0.65–4.99]

FLD, first line of drugs; SLD, second line of drug; *p < 0.05, **p < 0.001; reference category, unsuccessful outcomes; COR, crude odds ratio; AOR, adjusted odds ratio; CI, confidence interval; (Univariate analysis p < 0.15 is considered significant) multivariate model was significant, with chi square 37.03 DF 8, p < 0.0005; Hosmer–Lemeshow statistic chi square 4.73 (DF 7, N 116); collinearity (variance inflation factor = 10); and tolerance value > 0.10. SLD, second line of drug; ADEs, adverse drugs events.

### Frequency of Adverse Events

Out of 116 patients, 59 (50.8%) experienced at least one ADE. Most ADEs occurred during the 6-month interim period of MDR-TB treatment. Among all types of ADEs, nausea and vomiting (33%), arthralgia (28.4%), psychiatric disturbance (20.6%), and gastritis (10.3%) were mostly reported by patients during clinical follow-up assessment. Similarly, hearing disturbance (11.2%), gastritis (10.3%), skin rashes (6%), headache (4.3%), peripheral neuropathy (3.4%), and visual disturbance (1.7%) were also observed (Table 5).

**TABLE 5:** Prevalence and management of adverse events in patients with MDR-TB treatment.

Adverse drugs event	n% (116)	Temporarily discontinued/dose adjustment	Permanently discontinued
Gastrointestinal disorders	55 (47.4)	Managed symptomatically	Managed symptomatically
Gastritis	12 (10.3)	Managed symptomatically	Managed symptomatically
Nausea and Vomiting	36 (33)	PAS was stopped in patients for 15 days (*n* = 2)	Managed symptomatically
Diarrhea	3 (2.6)	PAS was stopped for 1 week as advised by the consultation (*n* = 2)	Managed symptomatically
Anorexia	4 (4.3)	Managed symptomatically	Managed symptomatically
Arthralgia	33 (28.4)	Temporarily stopped the Pyrazinamide for 2 weeks (*n* = 3)	Stopped pyrazinamide and revised regimen (*n* = 1)
		Dose reduction of pyrazinamide (*n* = 1)	
Psychiatric disturbances	24 (20.6)	Stopped cycloserine for 15 days (*n* = 3)	Cycloserine was stopped completely (*n* = 3)
		Dose reduction of cycloserine (*n* = 5)	
Peripheral neuropathy	4 (3.4)	Linezolid was stopped for 2 weeks (*n* = 2)	Managed symptomatically
		Pyridoxine dose was increased to 200 mg/day (*n* = 3)	
Headache	5 (4.3)	Managed symptomatically	Managed symptomatically
Skin rashes	7 (6%)	Reduced the dose of pyrazinamide (*n* = 1)	Managed symptomatically
		Replaced clofazimine with tablet linezolid (*n* = 1)	
Hearing disturbance	13 (11.2)	The frequency of amikacin was reduced (*n* = 3)	Amikacin shifted to capreomycin (*n* = 1)
Visual disturbance	2 (1.7)	Ethambutol was temporarily stopped, and clofazimine was added to the regime (*n* = 2)	Managed symptomatically

The individual could have more than one adverse drug event.

All the adverse events were managed by pharmacological, psychological, and supportive therapies. In addition, education was provided during each follow-up visit to patients regarding ADEs and their management. ADEs were thoroughly evaluated, and it was found that no drug(s) should be permanently discontinued unless they pose a life-threatening situation or causing a lasting injury to the patient. But in a worse condition, all the required changes were made according to the WHO guidelines. Among 11 (9.48%) MDR-TB patients, temporary medications were stopped, and drugs were successfully reintroduced within 4 weeks after the problem resolution. For 6 (5.1%) drug-resistant TB patients, permanent changes were made in the regimen, and for 14 (12.0%) patients dose adjustment was suggested during the study time. All of the problematic medicines that had been temporarily discontinued over the course of therapy were restarted after the ADRs had abated.

Symptomatic management was carried out for all the patients who had the gastrointestinal disorder until the problem was resolved. In case of persistent diarrhea, temporary discontinuation of para-amino salicylic acid for 1 week (*n* = 2) was advised by the consultant.

In case of no improvement or worsening of arthralgia, consultation with a physician withheld the suspected agent temporarily (*n* = 3) or dose of the offending drug (pyrazinamide) was reduced (*n* = 1) or permanently stopped (*n* = 1). In these four patients, pyrazinamide and clofazimine were replaced with ethambutol and ethionamide, respectively.

Psychiatric problems reported by drug-resistant TB patients compelled the healthcare team for modification in the treatment regimen. Cycloserine was temporary stopped in three patients, and for five patients, the dosage was reduced for 15 days, while in three patients cycloserine was completely stopped after proper consultation with a physician.

Similarly, in case of peripheral neuropathy, linezolid was stopped for 2 weeks (*n* = 2). In three patients with ototoxicity, frequency of amikacin was reduced, and in one patient, the regime was switched from amikacin daily to alternate day’s capreomycin, with physician’s consultation. In case of skin rash, the physician advised to reduce the dose of pyrazinamide and replace clofazimine with tablet linezolid for 15 days (1) Table 5.

### Patient Satisfaction

Out of 116 MDR-TB patients, 75 patients were available for patient satisfaction feedback regarding pharmacist-counseling assessment, and the majority (94.6%) of the respondents agreed that they were able to receive consultation without having any trouble with a pharmacist. A total of 81.3% agreed that they developed the knowledge related to MDR-TB according to their needs. However, 82.7% assured that the pharmacist helped resolve their medication-related queries during the treatment session. The majority (97.3%) appeared promising to recommend counseling from a pharmacist to others and recommended that this facility should be accessible in community pharmacies in the district. More than half of the patients (65.3%) were willing to pay for this counseling service. Moreover, 73.3% of the patients appeared satisfied with the counseling Table 6.

**TABLE 6:** Patient satisfaction feedback regarding pharmacist counseling.

Patient satisfaction feedback regarding pharmacist counseling	Response	n 75 (%)
Were you able to get counseling without any difficulty?	Yes	71 (94.6)
	No	4 (5.4)
Were you able to obtain the knowledge you required?	Yes completely	61 (81.3)
	Yes, to some extent	5 (6.7)
	No, I did not get	9 (12)
Did you find the pharmacist helpful in resolving your questions?	Very helpful	62 (82.7)
	Somewhat helpful	10 (13.3)
	Not helpful	3 (4)
What is your opinion about the time duration of pharmacist counseling?	More time should be given	28 (37.3)
	Appropriate time was given	45 (60)
	My time was wasted	2 (2.7)
Will you recommend getting counseling from pharmacists to others?	Yes	73 (97.3)
	No	2 (2.7)
In your opinion, should this service be offered by pharmacies in your locality?	Yes	73 (97.3)
	No	2 (2.7)
Are you willing to pay for this counseling service?	Yes	49 (65.3)
	No	26 (34.6)
How would you rate your satisfaction with pharmacist counseling?	Very satisfied	55 (73.3)
	Satisfied	12 (16)
	Uncertain	6 (8)
	Not satisfied	2 (2.7)

## Discussion

Multidrug TB treatment has long therapy duration (18 months to 2 years), and the number of drugs used for the treatment is comparatively more complex than that for drug-susceptible TB (Khan et al., 2022). Numerous side effects are identified that result in patients’ non-compliance and predictably poor outcomes (Tahaoğlu et al., 2001; Mukherjee et al., 2004). Therefore, monitoring and appropriate administration of drugs are of utmost significance for the successful treatment of MDR-TB patients (Zhang et al., 2017; Ahmad et al., 2018). In this current study, gastrointestinal disorders were the most common type of adverse events observed in patients (50.9%). The results of the present study are parallel to previously reported studies (Farazi et al., 2014; Javadi et al., 2007; Laghari et al., 2020). However, a study reported higher (71.3%) incidence of ADEs in MDR-TB patients. These differences between studies are likely due to the differences in patient characteristics, treatment regimens, and AE-monitoring approaches. In this study, ADEs were monitored prospectively following the patient-centered care approach delivered by the pharmacist. In our study, 50.9% of the patients developed ADEs of various severities during treatment, of whom 21.5% required minimal modification of the TB treatment regimen and symptomatic treatment in managing ADEs, and these results are consistent with previous studies (Javadi et al., 2007; Baghaei et al., 2011). While maximal modification in treatment was reported in few studies as compared with the current study, i.e., 55.2% from Turkey, 29% from Namibia, and 84% from Latvia (Sagwa et al., 2014; Shin et al., 2007; Törün et al., 2005). These findings point out the promising and important role of the pharmacist to manage all adverse events. All the adverse events in the study were managed by pharmacological, psychological, and supportive therapy. Those who developed gastrointestinal disorders were reassured and symptomatically managed by adding secondary drugs. Patients with adverse events such as nausea and vomiting were advised to perform liver function tests and take anti-TB medication after a light meal. To stop vomiting symptomatically, an antiemetic was added to their regimen. In the case of persistent diarrhea, para-amino salicylic acid was stopped for 1 week or until the adverse event was not resolved. Metronidazole was also prescribed by the physician for the relief of abdominal pain and diarrhea. In the current study, modification in patient’s regimes due to gastrointestinal disorders was according to the previously reported studies (Merid et al., 2019; Ahmad et al., 2018). In the current study, one of the reasons for the adverse events due to gastrointestinal disorders could be that 39.7% of patients were underweight, and they might not have tolerated the multidrug regimen. Previous studies also reported that underweight patients were more prone to ADEs (Laghari et al., 2020; Zhang et al., 2017). Therefore, appetite-stimulating agents and nutritional supplements were added to increase body weight in patients who were underweight.

A significant percentage of the patients (28.4%) experienced arthralgia, inconsistent with the results from India (Qureshi et al., 2007), Japan (Inoue et al., 1999), and Namibia (Sagwa et al., 2013). However, slightly less arthralgia is reported from China (Zhang et al., 2017). In the current study, both pyrazinamide and clofazimine were reported to be the leading cause of arthralgia. The findings of this study are in line with previous studies that reported pyrazinamide as a renowned cause of arthralgia in MDR-TB treatment (Farazi et al., 2014; Abraham et al., 2006). Symptoms of arthralgia were reduced by supportive and pharmacological treatment. The approach of treatment modification applied in our study was similar to the approach used by previous studies (Gülbay et al., 2006; Yang et al., 2017; Ahmad et al., 2018). Another adverse event, the psychiatric disturbance (20.6%), was almost parallel to the previous studies conducted in Turkey (21.3%) and Russia (20.5%) (Törün et al., 2005; Shin et al., 2007). The previous study also mentioned that the leading cause of the MDR-TB treatment modification was a psychiatric disturbance among patients. Cycloserine and ethionamide were recognized as the foremost drugs associated with psychiatric disorders (Leston et al., 1970; Yang et al., 2017; Zhang et al., 2017). Ototoxicity reported in our study (11.2%) was several times lower than that reported in the previous studies, viz., 55% reported in the United Kingdom (Arnold et al., 2017), and 14.5% reported in Iran (Baghaei et al., 2011)), while the cases of ototoxicity in the current study was more than that in the study conducted in Ethiopia (4.8%) (Shibeshi et al., 2019). The variability in the results might be due to the unavailability of proper audiometric assessments at regular intervals at the study site. This might have caused the under-reporting of the actual ototoxicity frequency in our study. Audiometric assessment with consistent intervals of follow-up visits must be considered in patients’ AE detection. The ototoxicity signs were recognized through assessment of hearing loss, imbalance, and visual disturbance. The prevalence of skin adverse reactions was comparatively lower than that reported in previous studies (Schaberg et al., 1996; Yee et al., 2003). We also observed 3.4% of neurologic side effects, much lower than that in a study conducted in Peru (36.7%) (Furin et al., 2001). Linezolid, which was temporarily discontinued, was reintroduced once symptoms resolved, and the dose of vitamin B6 was increased. Elderly patients are predominantly susceptible to ADRs. According to our results, the age group of above 46 years was more significantly associated with ADRs (OR 4.50; 95% CI 1.05-19.2 *p* < 0.05). The result of this study is entirely consistent with previous study reports (Laghari et al., 2020; Bezu et al., 2014). Geriatrics patients’ liver is less able to metabolize many drugs, and the kidneys are less able to eliminate the drug from the body (Merid et al., 2019). Most reported ADEs were also associated with the smokers’ group as compared with nonsmokers (OR 4.20; 95% CI 1.31-13.4). This result was supported by experimental and clinical studies that smoking increases the chance of ADEs (Bezu et al., 2014; Chung-Delgado et al., 2011). Bedaquiline is a novel drug approved by the WHO, for the treatment of MDR TB. This study investigated the safety of bedaquiline; patients who were without a bedaquiline regime have a higher chance of adverse events (OR 3.54; 95% CI 1.23-10.1) than the other group. The results of this study are consistent with previous studies (Gao et al., 2021; Mbuagbaw et al., 2019; Lan et al., 2020). This indicates that bedaquiline added to a background regimen can improve the rate of successful outcomes and quality of life.

Most patients looked for counseling with no trouble, discovered the pharmacists being helpful, seemed happy with the information they acquired, and looked for more pharmacist counseling opportunities. The findings of our study are consistent with other studies (Alqarni et al., 2019; Naqvi et al., 2019). A higher percentage of patients were willing to pay for the service, and the satisfaction score finding is in line with studies conducted in the United States and Saudi (Poulos et al., 2008; Alshayban et al., 2020). Pharmacists need to provide appropriate, clear, and relevant information to patients about their medications. The results of this study suggest the role of the pharmacist’s services in the Pakistan’s TB health sector. Therefore, efforts to emphasize pharmacists’ role, as a health promoter, will help attain safe and effective pharmaceutical care services.

There are several limitations in our study. First, we only evaluated patients from a single TB referral hospital that may have introduced selection bias; as a single-centered study, its results cannot be generalized. Second, the laboratory tests, especially audiometry, were not performed at the baseline level due to limited resources provided at the study site.

Despite these limitations, we believe that our study provides important information regarding the side effects of second-line anti-TB drugs in resource-poor settings.

## Conclusion

In conclusion, we believe that adverse drug events have implications not only for the patient but also for the entire healthcare system. In the present study, ADEs were highly prevalent with second-line anti-TB therapy, but neither led to permanent discontinuation of the regime nor significantly conceited the treatment outcomes. Current findings recommend that ADEs might be well managed by sympathetic, emotional, and pharmaceutical therapy and may be provided without disturbing anti-DR-TB regime. Bedaquiline coupled with other active medications reduce ADEs in MDR-TB patients. As a result, bedaquiline usage in DR-TB patients should be promoted. The elderly patients with active smoking behavior and those who have a delay in treatment initiation are more prone to ADEs. The contribution of clinical pharmacists in TB control programs may help caregivers and patients in the rational use of medication, early identification, and management of ADEs.

## Data Availability

The raw data supporting the conclusion of this article will be made available by the authors, without undue reservation.
